# Application of Numerical Analysis of the Shape of Electron Paramagnetic Resonance Spectra for Determination of the Number of Different Groups of Radicals in the Burn Wounds

**DOI:** 10.1155/2017/4683102

**Published:** 2017-05-02

**Authors:** Paweł Olczyk, Katarzyna Komosinska-Vassev, Paweł Ramos, Łukasz Mencner, Krystyna Olczyk, Barbara Pilawa

**Affiliations:** ^1^Department of Community Pharmacy, School of Pharmacy and Division of Laboratory Medicine in Sosnowiec, Medical University of Silesia, Kasztanowa 3, 41-200 Katowice, Poland; ^2^Department of Clinical Chemistry and Laboratory Diagnostics, School of Pharmacy with the Division of Laboratory Medicine in Sosnowiec, Medical University of Silesia, Jedności 8, 41-200 Katowice, Poland; ^3^Department of Biophysics, School of Pharmacy with the Division of Laboratory Medicine in Sosnowiec, Medical University of Silesia, Jedności 8, 41-200 Katowice, Poland

## Abstract

*Background.* The evidence exists that radicals are crucial agents necessary for the wound regeneration helping to enhance the repair process. *Materials and methods*. The lineshape of the electron paramagnetic resonance (EPR) spectra of the burn wounds measured with the low microwave power (2.2 mW) was numerically analyzed. The experimental spectra were fitted by the sum of two and three lines. *Results*. The number of the lines in the EPR spectrum corresponded to the number of different groups of radicals in the natural samples after thermal treatment. The component lines were described by Gaussian and Lorentzian functions. The spectra of the burn wounds were superposition of three lines different in shape and in linewidths. The best fitting was obtained for the sum of broad Gaussian, broad Lorentzian, and narrow Lorentzian lines. Dipolar interactions between the unpaired electrons widened the broad Gaussian and broad Lorentzian lines. Radicals with the narrow Lorentzian lines existed mainly in the tested samples. *Conclusions*. The spectral shape analysis may be proposed as a useful method for determining the number of different groups of radicals in the burn wounds.

## 1. Introduction

Burns are complex inflammatory or necrotic damages of both the layers and tissues exposed to the action of different energies which occur after exceeding the protective capabilities of the organism. The healing process of burn wounds with the loss of tissue and infection of the damaged place occurs by granulation. Healing of burn wounds is a complex, biologic process, based on the replacement of the damaged tissue with a living one [1, 2]. Restoring tissue integrity is the result of cell interactions (platelets, neutrophils, monocytes/macrophages, fibroblasts, endothelial cells, and keratinocytes) and such extracellular matrix (ECM) components as, fibronectin, glycosaminoglycans (GAGs), proteoglycans (PGs), thrombospondin, tenascin, vitronectin, and collagens [1, 2]. Regardless of the manner of the healing, it always consists of four subsequent stages: homeostasis, inflammation, proliferation (replication and synthesis), and remodeling [3, 4]. The key element of the burn wound healing is to achieve the balance between the processes of biosynthesis and the ECM components degradation. The essential role in the discussed process is played by matrix metalloproteinases (MMPs) which constitute a group dependent on endopeptidases' cycle synthesized in the cells and released in the extracellular space in an inactive form (proMMPs). The activity of the enzymes in question is precisely regulated on the transcription level [5–7], as well as in the postsynthetic modification of the mentioned proteases occurring with radicals. Moreover, radicals play an important role in the modification of extracellular components participating in the healing process, that is, glycosaminoglycans, collagens, and noncollagenous glycoproteins. As it has been shown in the study, the consequence of the influence of the thermal factor in the wound bed matrix filling in the damaged skin is the process of intensified synthesis of radicals. Thermal injury of the skin is a common traumatic oxidation injury that results in a local tissue damage and a systemic inflammatory response. Both of the abovementioned changes are related to the increased radical formation. As a result of the tissue damage, different sources of oxygen radicals become activated. The greatest source of reactive oxygen species and radicals in the burn injury is the xanthine oxidase-mediated reactions producing both superoxide radical and hydrogen peroxide during traumatic conditions [2–5]; however, the evidence exists that the neutrophil-mediated radical production may be more pronounced than xanthine oxidase in ischemia-reperfusion injury during burn tissue damage [8]. The role of oxygen radicals as causative agents in the local wound response as well as in distant organ injury is well documented [9]. Oxygen radicals take part in many metabolic processes, acting as harmful agents to proteins, DNA, and RNA causing deteriorative oxidative damage. In the early stages of the systemic inflammatory process in the course of burn healing, radicals exert their actions via the activation of such nuclear factors as NFkB or AP-1 and induce the local synthesis of inflammatory cytokines such as TNF*α*, Il-1b, Il-6, Il-8, and Il-10 [10, 11]. Radicals also act as important mediators of the systemic inflammatory response. The evidence exists that radicals are necessary for the proper healing of skin wounds helping to enhance the repair process [12]. However, the role of radicals in burn wound repair is still little explored.

The numerical analysis of the lineshape of the electron paramagnetic resonance (EPR) spectra was performed to find different groups of radicals in the burn wounds. Different types of thermally formed radicals in skin and tissue revealed characteristic EPR signals, which were identified. The aim of this study was the application of numerical procedures to multicomponent microwave absorption curves to obtain information about the number of different groups of radicals in the burn wounds. This work was the continuation of our earlier comparative EPR studies of different groups of radicals in the burn wounds treated with the propolis and silver sulphadiazine salt [13]. The advanced modern numerical procedures were used.

## 2. Experimental

### 2.1. Samples

The study protocol was approved by the Regional Ethics Committee for Experiments on animals of the Medical University of Silesia. The experiments were conducted in the Central Experimental Animal Quarters of the Medical University of Silesia. The Polish Landrace pigs were kept in uniform zoohygienic conditions both before and during the experiment. The animals were fed with a balanced mixture R 233 in the full physical and mental comfort eliminating the stress reactions. The skin burn wounds were inflicted according to Hoekstra's model [14]. In the study, three 16-week sows weighing about 35-40 kg were used. After the former premedication (0.06 mg/kg bw atropine sulfate sc., 1 mg/kg bw ketamine hydrochloride iv., and 1 mg/kg bw xylazine hydrochloride iv.), the pigs were subject to general anesthesia with 5 mg/kg bw thiopental sodium salt iv. After achieving deep analgesia, each animal was inflicted with burn wounds sized 1.5 cm × 3 cm by applying for 20 seconds lancerton D with an electrode preheated to 170°C (in symmetrical gaps, 9 on both sides). The study material was constituted by tissue samples (collected in triplicates) from the area filling the wounds on the 3rd day after the thermal damages. The obtained tissue material was frozen and stored in the temperature of −75°C until the biochemical analysis. At the beginning of the study, the biologic material was technically treated and preliminarily disintegrated and weighed. The prepared tissue samples were homogenized in acetone with a mechanical homogenizer (30,000 RPM, 4°C, 30 minutes) until obtaining a homogenous suspension. The abovementioned procedure allowed a simultaneous degreasing of the samples. The tissue homogenates were dried to constant mass and weighed again [14].

### 2.2. EPR Measurements

The EPR spectra of the burn wounds located in the thin-walled glass tubes with the external diameter of 3 mm were measured at room temperature by the use of an X-band (9.3 GHz) electron paramagnetic resonance spectrometer of the Radiopan Firm (Poznań, Poland) with magnetic modulation of 100 kHz. The mass of the samples in the tubes was obtained by weight of Sartorius Firm (Germany). The mass of the burn wounds samples was calculated as the difference between the mass of the tube with the sample and the mass of the empty tube. The high-paramagnetic purity characterized the tubes and they were of the EPR signals in the measurement conditions.

The total microwave power produced by klystron in the microwave bridge of the EPR spectrometer was 70 mW. The EPR spectra were recorded without microwave saturation effects with the low microwave power equal to 2.2 mW. This low value of microwave power corresponded to the high value of attenuation equaling 15 dB, according to the formula [15, 16]

(1) $$\begin{array}{c} attenuation\;\left[dB\right]=10\;\mathrm{lg}\left(\frac{M_{o}}{M}\right), \end{array}$$

where *M_o_* is the total produced microwave power (70 mW) and *M* is the microwave power used during the EPR measurement. Microwave frequency (*ν*) was directly measured by MCM101 recorder produced by EPRAD Firm (Poznań, Poland).

The EPR spectra were measured as the first derivative of microwave absorption. The Rapid Scan Unit of Jagmar Firm (Kraków, Poland) was used as the data acquisition system. The professional spectroscopic program of the Jagmar Firm and LabView program (USA) was applied.

### 2.3. The Numerical Analysis of the Lineshape of the EPR Spectra

The lineshape of the EPR spectra of the burn wounds were numerically analyzed. The spectra were fitted by single lines and by the sum of two and three lines. The lines with Gaussian and Lorentzian shapes were used. The experimental EPR spectra of the burn wounds were fitted by theoretical lines as a superposition of two Gaussian lines (GG), two Lorentzian lines (LL), and by the sum of Gaussian and Lorentzian (GL) lines. The experimental spectra were fitted by the sum of three Gaussian Lines (GGG), three Lorentzian lines (LLL), two Gaussian lines and one Lorentzian line (GGL), and one Gaussian and two Lorentzian lines (GLL). The best fitting of the experimental EPR spectra of the burn wounds was recognized as the one giving the lowest value of the standard deviation (*S*).

The linewidths (Δ*B*_pp_) and the percentage fractions of each component's line in the total EPR spectrum were determined. The fraction of the individual lines in the total spectrum was obtained as the percentage fraction of the integral intensity of these component's lines in the integral intensity of the total spectrum.

The numerical procedure of the spectroscopic analysis is described below. The analysis of the complex signal, which is given as a time series *f*_*r*_(*x*), *x* = 1*. N* represents a discrete set of function values within domain (−5, 5) and time step ∆*x* = 10^−3^. The best match of *f*_*r*_ function using *f*_*p*_ function defined as a composition of basic functions was searched. The first derivative of microwave absorption function for radicals was taken into account. The signal was filtered before approximation and its reference point (0,0) was defined.

Filtering of the signal was performed by moving an average filter and the filter based on fast Fourier transformation (FFT) [17, 18]. The signal changes were minimized by the use of the Fourier filter based on the frequency domain. The filter removes the frequencies with the amplitude value below the threshold of 0.05. The strongest signal was observed for the lowest frequencies. The filter removes the signal above 15 Hz.

The reference point (0,0) was found by defining a horizontal and vertical shift of a filtered signal *f*_*r*_ according to *x*- (by *dx* value) and *y*- (by *dy* value) axes. To find the reference values, the following rules were used: (a) the sum of values of samples separated by *x*-axis is close to 0, and (b) the absolute value of the sum of function from left side of *y*-axis plus the absolute value of the sum of function from the right side of *y* were maximized. Finally, the filtered and shifted *f*_*r*_ function is processed by the approximation algorithm. The Gaussian and Lorentzian functions were selected to the approximations. The genetic algorithm with [19] the conjugate gradient method was used [20].

## 3. Results and Discussion

Radicals and other paramagnetic molecules play an important role in both physiological and pathological conditions. Radicals are formed in burn wound matrix in thermolysis processes [21]. As a result of high-temperature effects, the chemical bonds are broken in the molecules of skin structures and, as a result, the molecules with unpaired electrons appear [22]. It is expected that different types of radicals are formed in the skin. Because of the contents of unpaired electrons, these molecules or molecular fragments are reactive and unstable units. They may cause major biochemical reactions in the skin and in the neighboring tissues [23]. These molecules can also be a pivotal factor mediating systemic disturbances characterizing burn injury [24]; however, the exact role and impact of radicals formed during thermal skin injury still remains unclear.

Different groups of radicals in the skin burn wounds have been found in our study.

**Figure 1:**
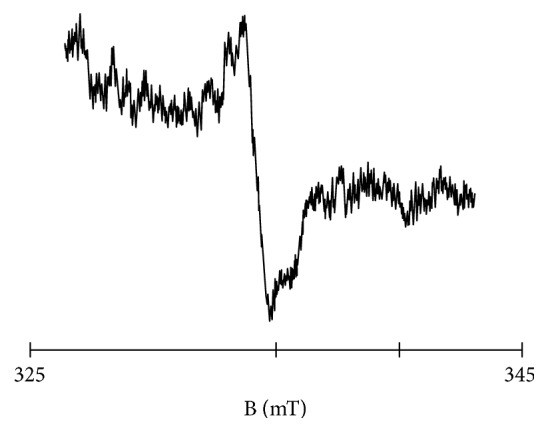
The first-derivative experimental EPR spectrum of the burn wound measured with the low microwave power of 2.2 mW. B—magnetic induction.

**Figure 2:**
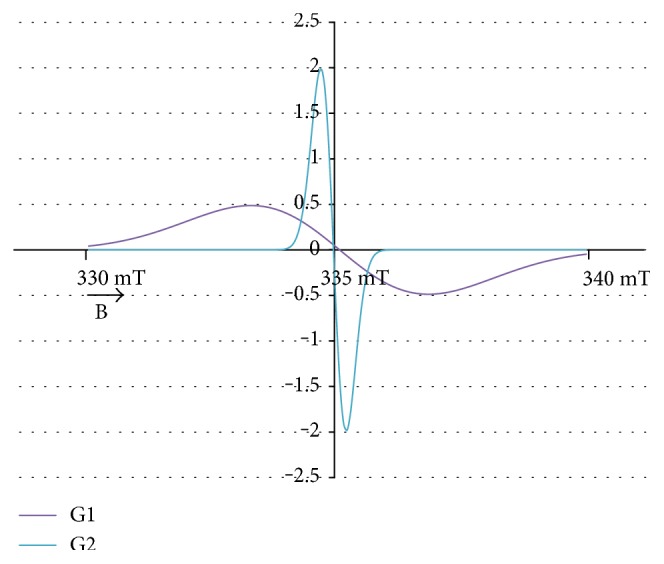
The component lines of the experimental EPR spectrum of the burn wounds for its numerical fitting by the sum of two Gaussian lines (GG). G—line of Gaussian shape. B—magnetic induction.

**Figure 3:**
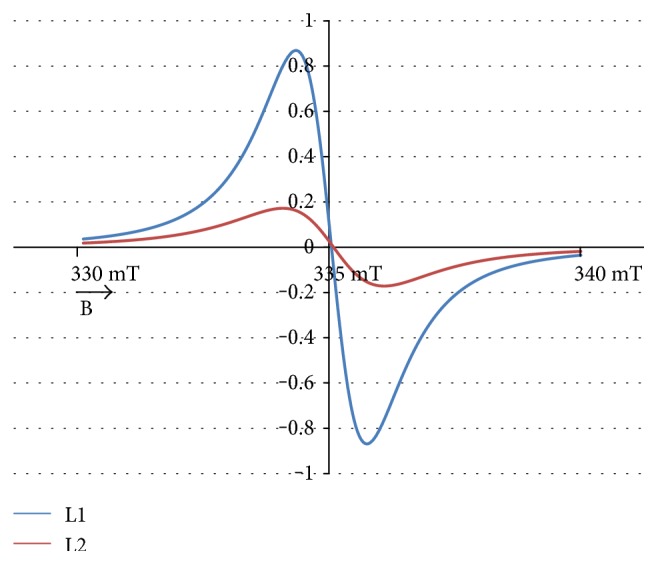
The component lines of the experimental EPR spectrum of the burn wounds for its numerical fitting by the sum of two Lorentzian lines (LL). L—line of Lorentzian shape. B—magnetic induction.

**Figure 4:**
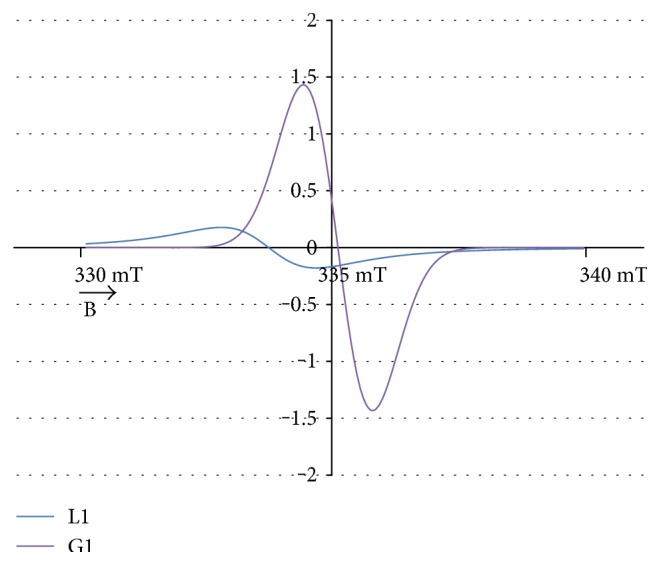
The component lines of the experimental EPR spectrum of the burn wounds for its numerical fitting by the sum of one Gaussian and one Lorentzian lines (GL). G—line of Gaussian shape. L—line of Lorentzian shape. B—magnetic induction.

**Figure 5:**
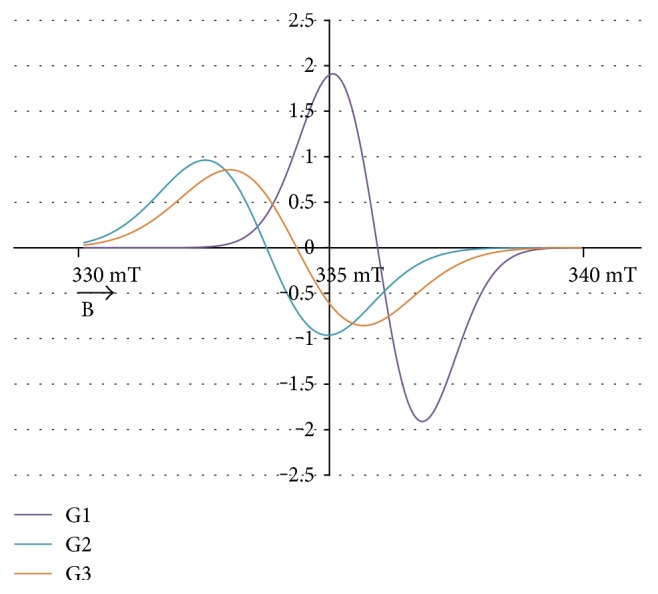
The component lines of the experimental EPR spectrum of the burn wounds for its numerical fitting by the sum of three Gaussian lines (GGG). G—line of Gaussian shape. B—magnetic induction.

**Figure 6:**
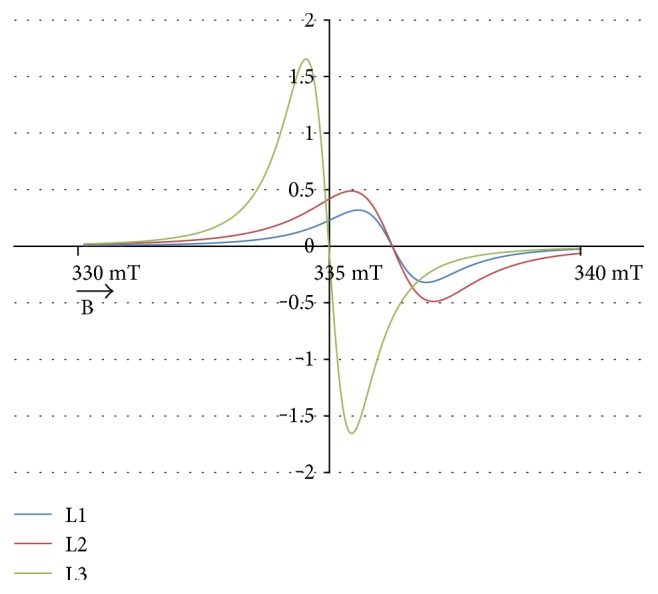
The component lines of the experimental EPR spectrum of the burn wounds for its numerical fitting by the sum of three Lorentzian lines (LLL). L—line of Lorentzian shape. B—magnetic induction.

**Figure 7:**
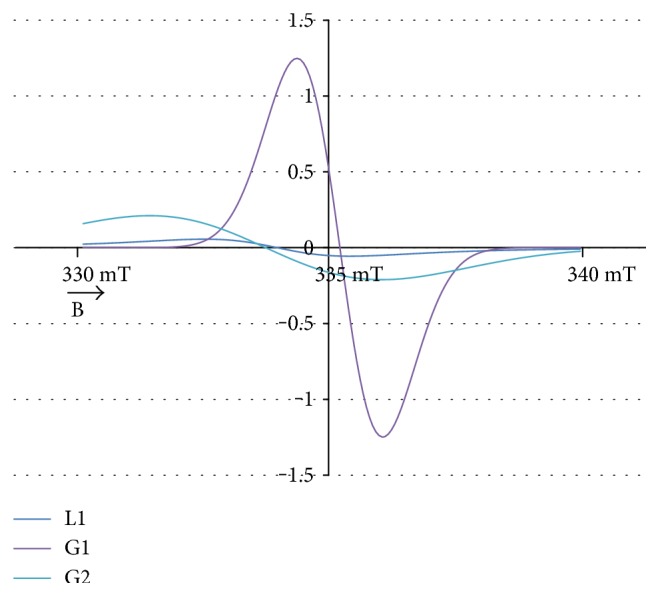
The component lines of the experimental EPR spectrum of the burn wounds for its numerical fitting by the sum of two Gaussian and one Lorentzian lines (GGL). G—line of Gaussian shape. L—line of Lorentzian shape. B—magnetic induction.

**Figure 8:**
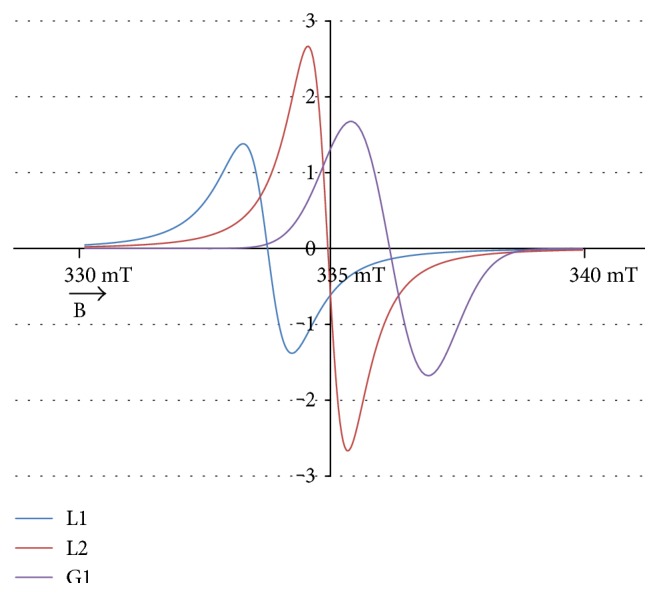
The component lines of the experimental EPR spectrum of the burn wounds for its numerical fitting by the sum of one Gaussian and two Lorentzian lines (GLL). G—line of Gaussian shape. L—line of Lorentzian shape. B—magnetic induction.

**Figure 9:**
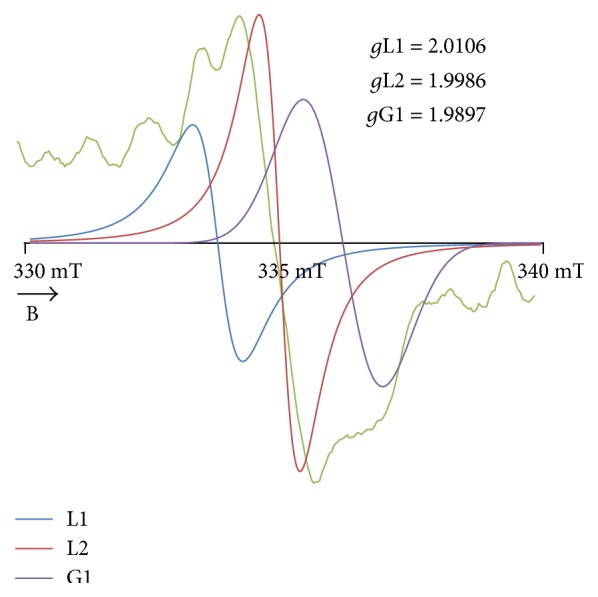
The complex resultant EPR spectrum after reduction of the noise and their Gaussian (G) and Lorentzian (L) component lines. B—magnetic induction. *g*L1, *g*L2, and *g*G1—*g* factors for the component lines.

The experimental EPR spectrum of the burn wounds is shown in Figure 1. The complex shape of this spectrum indicated that it was a superposition of several component lines. The results of the theoretical deconvolution of this spectrum by theoretical two or three component curves are presented in Figures 2–8. Two component lines for fitting by the sum of two Gaussian lines (GG), two Lorentzian lines (LL), and sum of one Gaussian and one Lorentzian lines (GL) are shown in Figures 2–4, respectively. Three component lines for fitting by the sum of three Gaussian lines (GGG), three Lorentzian lines (LLL), the sum of two Gaussian and one Lorentzian lines (GGL), and the sum of one Gaussian and two Lorentzian lines (GLL) are shown in Figures 5–9, respectively.

The numerical analysis of the spectral shape pointed out that the experimental EPR spectrum of the burn wounds was a superposition of the three lines (Tables 1 and 2). The best fitting was obtained for the sum of one Gaussian (G1) line and two Lorentzian (L1, L2) lines (Table 2, Figure 8). Three different groups of radicals existed in the tested samples. These radicals were responsible for Gaussian (G1), Lorentzian (L1), and Lorentzian (L2) lines. It indicated that the tested burn wounds contained three tissue structures with a different resistance to the thermal factor. The high-temperature disrupted chemical bonds in these structures and major radical systems occurred. Unpaired electrons were located in the units consisted of different molecules. Usually, the high thermal resistance characterizes large organic aromatic structures formed by the multiaromatic rings [25–27]. The aliphatic and the simple aromatic units usually revealed a very low thermal stability [25–27]. It seems that radicals in the burn wounds were formed by thermal decomposition of all the mentioned chemical structures: large aromatic, simple aromatic, and aliphatic units. All these chemical units existed in tissues [26, 27]. The advantages of the proposed numerical spectral analysis consisted in the resolution of the component signals in the complex EPR spectra to determine the number of the group of radicals in the tested thing. The analysis of the changes of the asymmetry parameters of the EPR spectra with the increase of the microwave power confirmed their multicomponent character without the deconvolution to the components [15, 16, 28–33].

A different chemical origin of each of the group of radicals was reflected in linewidths (Δ*B*_pp_) of their EPR signals. The parameters of the EPR lines of these groups of radicals are visible in Table 2. The EPR spectrum was deconvoluted to the broad Gaussian (G1) and the broad (L2) and the narrow Lorentzian (L1) lines. The broadest EPR component was the Gaussian (G1) line. Strong dipolar interactions between the unpaired electrons of radicals broadened the EPR component (G1) with Gaussian shape. The broadening dipolar interactions were stronger between the unpaired electrons of radicals with the broadest Gaussian (G1) EPR line than between the radicals with the narrower Lorentzian (L1, L2) line. The relatively stronger dipolar broadening was observed for the broader Lorentzian (L1) line than for the narrower Lorentzian (L2) line. In other works [34–36], the broad EPR lines for unpaired electrons in multiring carbon organic materials were measured, while the narrow EPR lines were detected for aliphatic and simple aromatic units in carbon materials.

The amplitudes of the EPR components decrease in the following order: broad Gaussian (G1) line > narrow Lorentzian (L2) line > broad Lorentzian (L1) line (Table 2). The highest content of the radicals with the narrow Lorentzian (L2) line (41.2%) characterized the examined burn wounds (Table 2). The lowest content of the radicals with the broad Lorentzian (L1) line (26.1%) was found for the tested burn wounds (Table 2). The content of the radicals with the broad Gaussian (G1) line was 32.1% (Table 2). Radicals with the narrow Lorentzian (L2) lines are likely to play an important role in the regeneration process of the burn wounds. These radicals revealed unpaired electrons in the larger aromatic units and probably they will interact with oxygen, oxygen molecules O_2_, and reactive oxygen species. It was proved for organic materials that radicals of such units actively interact with oxygen [34]. These radicals probably will protect the thermally treated tissues from the destruction by reactive oxygen compounds.

This work was a fine example of applying the electron paramagnetic resonance and the numerical procedures of the spectral lineshape analysis for identification of the complex radical system in tissues. The analysis of the total EPR spectra gives only information about the whole radicals system. The performed deconvolution of the resultant EPR spectra to the component lines allowed us to different groups of radicals in the biological samples.

Our findings have significant implications since the healing of the burn wound remains a challenge to modern medicine. Oxidative stress could contribute to secondary tissue impairment and altered immune function after burn traumatic injury. Moreover, the study indicated various groups of radicals arising during the healing process of burn wounds which may modulate the transition of ECM components as well as influence the activity of the components participating in degradation by matrix metalloproteinases, thus contributing to controlling the healing process at cellular level and conditioning the formation of the favorable biochemical environment favoring an effective tissue repair [13, 25]. Due to the important implications of radical in burn injury, further studies are needed to characterize the antioxidative defense mechanisms protecting tissues against oxidative damage. Extending biochemical studies concerning the antioxidative activity would be a valuable complement of studies assessing the burn wound healing process which would trigger the preparation of new methods of burn therapy.

## 4. Conclusions

The numerical study of the lineshape of the complex EPR spectra was a helpful method for identification of the number of different groups of radicals in the burn wounds. These EPR spectra were superposition of three lines: broad Gaussian (G1) line, broad Lorentzian (L1) line, and narrow (L2) Lorentzian line. The modern spectral analysis was proposed. This method additionally gave information about the percentage contents of each type of radicals in the burn wounds. Radicals with weak dipolar interactions responsible for the narrow Lorentzian (L2) line existed mainly in the examined burn wounds.

## Figures and Tables

**Table 1:** The parameters of the component lines of the EPR spectra of the burn wounds fitted by two (GG, LL, and GL) lines. G—Gaussian line. L—Lorentzian line. *A*—amplitude, Δ*B*_pp_—linewidth. *S*—standard deviation for the fitting.

Function	Parameters	L1	L2	L3	G1	G2	G3	*S*
GG	*A* (a.u.)	0	0	0	**0.97**	**3.95**	0	**202.8**
	Δ*B*_pp_ (mT)	0	0	0	**3.55**	**0.50**	0	
	Signal power (%)	0	0	0	**62.7**	**37.3**	0	

LL	*A* (a.u.)	**1.74**	**0.34**	0	0	0	0	**245.2**
	Δ*B*_pp_ (mT)	**1.37**	**2.06**	0	0	0	0	
	Signal power (%)	**78.9**	**21.1**	0	0	0	0	

GL	*A* (a.u.)	**0.36**	0	0	**2.86**	0	0	**249.5**
	Δ*B*_pp_ (mT)	**1.87**	0	0	**1.37**	0	0	
	Signal power (%)	**19.7**	0	0	**80.3**	0	0	

**Table 2:** The parameters of the component lines of the EPR spectra of the burn wounds fitted by three (GGG, LLL, GGL, and GLL) lines. G—Gaussian line. L—Lorentzian line. *A*—amplitude, Δ*B*_pp_—linewidth. *S*—standard deviation for the fitting.

Function	Parameters	L1	L2	L3	G1	G2	G3	*S*
GGG	*A* (a.u.)	0	0	0	**3.82**	**1.92**	**1.71**	**243.3**
	Δ*B*_pp_ (mT)	0	0	0	**1.81**	**2.37**	**2.69**	
	Signal power (%)	0	0	0	**42.5**	**29.1**	**28.4**	

LLL	*A* (a.u.)	**0.64**	**0.98**	**3.31**	0	0	0	**146.3**
	Δ*B*_pp_ (mT)	**1.37**	**1.62**	**0.87**	0	0	0	
	Signal power (%)	**15.6**	**28.1**	**56.3**	0	0	0	

LGG	*A* (a.u.)	**0.11**	0	0	**2.5**	**0.42**	0	**149.8**
	Δ*B*_pp_ (mT)	**2.87**	0	0	**1.68**	**4.62**	0	
	Signal power (%)	**6.5**	0	0	**67.2**	**26.3**	0	

LLG	*A* (a.u.)	**2.76**	**5.32**	0	**3.35**	0	0	**120.8**
	Δ*B*_pp_ (mT)	**1.0**	**0.75**	0	**1.56**	0	0	
	Signal power (%)	**26.1**	**41.2**	0	**32.1**	0	0	

## References

[1] Dulmovits B. M., Herman I. M. (2012). Microvascular remodeling and wound healing: a role for pericytes. *The International Journal of Biochemistry & Cell Biology*.

[2] Schultz G. S., Davidson J. M., Kirsner R. S., Bornstein P., Herman I. M. (2011). Dynamic reciprocity in the wound microenvironment. *Wound Repair and Regeneration*.

[3] Pazyar N., Yaghoobi R., Rafiee E., Mehrabian A., Feily A. (2014). Skin wound healing and phytomedicine: a review. *Skin Pharmacology and Physiology*.

[4] Guo S., DiPietro L. A. (2010). Factors affecting wound healing. *Journal of Dental Research*.

[5] Bourboulia D., Stetler-Stevenson W. G. (2010). Matrix metalloproteinases (MMPs) and tissue inhibitors of metalloproteinases (TIMPs): positive and negative regulators in tumor cell adhesion. *Seminars in Cancer Biology*.

[6] Cox T. R., Erler J. T. (2011). Remodeling and homeostasis of the extracellular matrix: implications for fibrotic diseases and cancer. *Disease Models & Mechanisms*.

[7] Gibson D. J., Schultz G. S. (2013). Molecular wound assessments: matrix metalloproteinases. *Advances in Wound Care*.

[8] Hansbrough J. F., Wikström T., Braide M. (1996). Neutrophil activation and tissue neutrophil sequestration in a rat model of thermal injury. *Journal of Surgical Research*.

[9] Ward P. A., Till G. O. (1990). Pathophysiologic events related to thermal injury of skin. *The Journal of Trauma*.

[10] Horton J. W. (2003). Free radicals and lipid peroxidation mediated injury in burn trauma: the role of antioxidant therapy. *Toxicology*.

[11] Farina J. A., Rosique M. J., Rosique R. G. (2013). Curbing inflammation in burn patients. *International Journal of Inflammation*.

[12] Xu S., Chisholm A. D. (2014). *C. elegans* epidermal wounding induces a mitochondrial ROS burst that promotes wound repair. *Developmental Cell*.

[13] Olczyk P., Ramos P., Komosinska-Vassev K., Stojko J., Pilawa B. (2013). Positive effect of propolis on free radicals in burn wounds. *Evidence-Based Complementary and Alternative Medicine*.

[14] Hoekstra M. J., Hupkens P., Dutrieux R. P., Bosch M. M. C., Brans T. A., Kreis R. W. (1993). A comparative burn wound model in the New Yorkshire pig for the histopathological evaluation of local therapeutic regimens: silver sulfadiazine cream as a standard. *British Journal of Plastic Surgery*.

[15] Wertz J. E., Bolton J. R. (1986). *Electron Spin Resonance Theory and Practical Applications*.

[16] Eaton G. R., Eaton S. S., Salikhov K. M. (1998). *Foundations of Modern EPR*.

[17] Wang D. Q. (2011). Least squares-based recursive and iterative estimation for output error moving average systems using data filtering. *Control Theory & Applications*.

[18] Wang S., Inkol R. J., Rajan S., Patenaude F. (2010). Detection of narrow-band signals through the FFT and polyphaser FFT filter banks: noncoherent versus coherent integration. *IEEE Transactions on Instrumentation and Measurements*.

[19] Chan R. R., Sudhoff S. D. (2010). An evolutionary computing approach to robust design in the presence of uncertainties. *IEEE Transactions on Evolutionary Computation*.

[20] Dong J., Jiao B., Chen L. A new hybrid HS-DY conjugate gradient method.

[21] Shimizu S., Tanaka H., Sakaki S., Yukioka T., Matsuda H., Shimazaki S. (2002). Burn depth affects dermal interstitial fluid pressure, free radical production, and serum histamine levels in rats. *The Journal of Trauma*.

[22] Park B. H., Saxer C., Srinivas S. M., Nelson J. S., de Boer J. F. (2001). In vivo burn depth determination by high-speed fiber-based polarization sensitive optical coherence tomography. *Journal of Biomedical Optics*.

[23] Portugal M., Barak V., Ginsburg I., Kohen R. (2007). Interplay among oxidants, antioxidants, and cytokines in skin disorders: present status and future considerations. *Biomedicine & Pharmacotherapy*.

[24] Latha B., Babu M. (2001). The involvement of free radicals in burn injury: a review. *Burns*.

[25] Olczyk P., Mencner Ł., Komosinska-Vassev K. (2014). The role of the extracellular matrix components in cutaneous wound healing. *BioMed Research International*.

[26] Rozancew E. G., Szolle W. D. (2004). *Organic Chemistry of Free Radicals*.

[27] Bartosz G. (2004). *The Second Face of Oxygen*.

[28] Stankowski J., Graja A. (1972). *Introduction to Quantum Electronics*.

[29] Morrish A. H. (1970). *Physical Basics of Magnetism*.

[30] Weil J. A., Bolton J. R. (2007). *Electron Paramagnetic Resonance: Elementary Theory and Practical Applications*.

[31] Ramos P., Pilawa B. (2010). The EPR examination of free radicals formation in thermally sterilized β-lactam antibiotics. *Current Topics in Biophysics*.

[32] Ramos P., Pilawa B. (2015). EPR examination of free radicals thermally formed in vaselinum flavum. *Nukleonika*.

[33] Ramos P., Pilawa B. (2015). Free radicals in thermally sterilized acidum boricum and optimization of this process. *Acta Poloniae Pharmaceutica-Drug Research*.

[34] Pilawa B., Więckowski A. B. (1997). Comparative EPR analysis of interactions between macerals and atmospheric oxygen. *Fuel*.

[35] Pilawa B., Więckowski A. B. (2007). Groups of paramagnetic centres in coal samples with different carbon contents. *Research on Chemical Intermediates*.

[36] Pilawa B., Więckowski A. B., Pietrzak R., Wachowska H. (2001). EPR studies of demineralized and oxidized coal. *Molecular Physics Reports*.

